# Verification of a Fluid–Structure Interaction Model for Aortic Stenosis Through Comparison With In Vitro Experiments

**DOI:** 10.1002/cnm.70169

**Published:** 2026-03-31

**Authors:** Sabine Verstraeten, Roel Meiburg, Koen Janssens, Martijn Hoeijmakers, Nils Karajan, Marcel Rutten, Marcel van't Veer, Pim Tonino, Frans van de Vosse, Wouter Huberts

**Affiliations:** ^1^ Department of Biomedical Engineering Eindhoven University of Technology Eindhoven the Netherlands; ^2^ ANSYS, Part of Synopsys Eindhoven the Netherlands; ^3^ DYNAmore GmbH, Part of Synopsys Stuttgart Germany; ^4^ Department of Cardiology Catharina Hospital Eindhoven the Netherlands; ^5^ Computational Science Lab Faculty of Science Institute for Informatics University of Amsterdam Amsterdam the Netherlands

**Keywords:** aortic stenosis, clinical decision support, fluid–structure interaction simulations, in vitro experiments, verification

## Abstract

Aortic stenosis (AS) severity is typically assessed by measuring pressure drop across the aortic valve in rest. However, this flow‐dependent metric is influenced by patients' cardiac function, complicating clinical decision‐making on valve replacement. Moreover, assessment during rest does not reflect valvular dynamics under higher flow rates (e.g., during exercise), and may underestimate severity in moderate AS patients. Patient‐specific Fluid–Structure Interaction (FSI) modelling offers a promising, non‐invasive method to simulate valve dynamics under varying conditions, independent of pressure exerted by the left ventricle. Therefore, this study aimed to experimentally verify an aortic stenosis FSI model using a patient‐specific aortic valve geometry, including calcifications, across flow conditions ranging from rest to exercise. To achieve this goal, in vitro experiments were conducted using a mock‐loop circulatory system with patient‐specific silicone rubber valve models, both calcified and non‐calcified. These experiments were replicated in FSI simulations. Overall, good agreement was observed between simulated and experimental results for the non‐calcified valve in terms of mean transvalvular flow (5% error on average) and aortic valve area (AVA) (10% error on average). Discrepancies were more pronounced in the calcified valve due to added complexity and uncertainty introduced by calcifications (8% and 7% error on average for mean transvalvular flow and AVA, respectively). For clinical implementation, two key challenges remain: (1) developing efficient and reliable methods to estimate valve leaflet material properties and pre‐stress, and (2) verifying the model against a broader range of in vitro data, followed by validating it against real, clinical data. Despite the remaining challenges, this study demonstrated the feasibility of using FSI models as a complementary, non‐invasive tool to assess AS severity and support clinical decision‐making on valve replacement.

## Graphical abstract

A patient‐specific Fluid–structure Interaction (FSI) model of aortic stenosis was verified against in vitro experiments using a mock‐loop circulatory system with patient‐specific calcified and non‐calcified valve models under varying flow conditions. The in vitro experiments were replicated in the FSI model. Good overall agreement was observed between simulations and experiments.

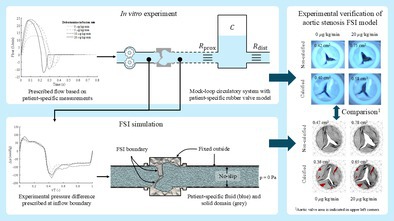

## 1 Introduction

Aortic stenosis (AS) is a progressive narrowing of the aortic valve, affecting approximately 2% of individuals over the age of 65 years [1]. Severity is typically assessed by echocardiography using mean transvalvular pressure drop ($\Delta p_{\text{mean}}$), peak transvalvular velocity ($V_{\max}$), and aortic valve area (AVA). According to current clinical guidelines, only patients with severe AS, defined by $\Delta p_{\text{mean}}$ > 40 mmHg, $V_{\max}$ > 4 m/s, and AVA < 1 cm^2^, are treated with valve replacement [2]. However, these metrics are strongly dependent on the patient's flow status, that is, the flow generated by the patient's left ventricle (LV). Low‐flow, low‐gradient (LF‐LG) AS patients for example, exhibit $\Delta p_{\text{mean}}$ < 40 mmHg, indicating non‐severe stenosis, with AVA < 1 cm^2^, indicating severe stenosis. In these patients, the reduced AVA may not necessarily reflect true stenosis. Instead, it could result from insufficient flow generated by the LV to fully open the valve. This condition is referred to as pseudo‐severe AS [3]. To differentiate between true‐severe and pseudo‐severe AS, a dobutamine stress echocardiography (DSE) is recommended. Dobutamine is a drug, that increases heart rate and contractility, thereby increasing flow. During DSE, a persistent AVA < 1 cm^2^ and rising Δp > 40 mmHg, indicate true‐severe AS. Conversely, normalisation of AVA (> 1 cm^2^) suggests pseudo‐severe AS [2]. In the latter, valve replacement is unlikely to be beneficial, since the underlying problem is left ventricular dysfunction rather than the valvular obstruction. Unfortunately, 30% of the DSE examinations fail, due to insufficient flow reserve, leaving the severity undetermined, which is a major limitation of the current clinical guidelines.

In addition to this limitation, Johnson et al. [4], have raised concerns about the restriction of DSE in the current guidelines to only a small subset of patients, namely patients with LF‐LG, with reduced LV ejection fraction. In their study they found heterogeneous relationships between pressure and flow across stenotic valves, which cannot be predicted from resting‐state measurements alone. Therefore, they proposed the stress aortic valve index (SAVI), which is the ratio between the time‐averaged systolic ejection pressure in the aorta and the time‐averaged systolic ejection pressure in the left ventricle, during dobutamine induced peak stress. In a follow‐up study, Zelis et al. [5], applied SAVI to patients with moderate aortic stenosis patients (AVA > 1 cm^2^, and Δp < 40 mmHg) and found that 39% of these patients demonstrated SAVI values, similar to those of patients with severe AS. These findings raise the hypothesis that current guidelines may overlook a subset of moderate AS patients who could potentially benefit from valve replacement, but are not considered for DSE in the current clinical workflow. While SAVI addresses an important gap, namely the under diagnosis of patients with moderate AS at rest, it still relies on dobutamine induced stress testing, and invasive pressure measurements. Although dobutamine induced stress testing is a relatively safe procedure, patients can still experience some reaction to the dobutamine infusion, such as nausea, headache, palpitations, and shortness of breath [6].

In summary, the current clinical workflow for AS severity assessment heavily relies on the patient's cardiac function, which can complicate clinical decision‐making. Additionally, assessments performed solely at rest may underestimate severity in patients with moderate AS. These limitations highlight the need for an alternative, non‐invasive approach that can assess valve function independently of cardiac function and under varying flow conditions, from rest to exercise.

A promising approach to address the limitations in the current AS severity assessment is the use of patient‐specific finite element analysis (FEA) models. These models have the potential to simulate valve dynamics under varying conditions, from rest to exercise, regardless of the pressure generated by the patient's LV, and in a non‐invasive manner. Previous studies have applied FEA to model aortic valves [7, 8], typically by applying pressure loads directly to the valve leaflets and focusing solely on mechanical outcomes such as stress, strain, and AVA. Borowski et al. [9] compared purely structural FEA with Fluid–Structure Interaction (FSI) simulations, which incorporate the influence of fluid dynamics. They found that omitting fluid dynamics significantly altered the predicted valve opening behaviour. Moreover, structural FEA cannot estimate clinically relevant metrics such as transvalvular flow, pressure drop, and SAVI that are essential for severity assessment. In contrast, FSI models can simulate transvalvular haemodynamics under varying flow conditions, enabling estimation of these relevant metrics. As such, FSI models have the potential to distinguish true from pseudo‐severe AS. Moreover, they can be used to non‐invasively assess severity in moderate AS under simulated stress conditions, helping to overcome underestimation of AS severity in these patients.

The use of FSI models to simulate aortic valve dynamics has been extensively studied in the past [10]. Early studies introduced two‐dimensional FSI frameworks to simulate healthy aortic valve behaviour [11, 12]. These were later extended to 3D simulations [9, 13, 14, 15, 16]. All these models were based on idealised and healthy aortic valve geometries with either no calcifications or with homogenously distributed calcifications. For clinical decision‐making, however, it is essential to simulate patient‐specific valve geometries that include realistic calcification morphology.

Govindarajan et al. [17] developed a patient‐specific FSI simulation framework that included geometries of the left ventricle, aortic valve, and ascending aorta, derived from CT imaging. LV wall motion was prescribed based on the patient's heart rate to generate physiological pressure conditions and simulate valve opening. They compared the fluid dynamics in the patient‐specific valve with and without calcifications and demonstrated that calcifications strongly influenced fluid dynamics (turbulent in calcified leaflets and laminar to turbulent in uncalcified leaflets). These findings indicate the importance of considering calcifications in such patient‐specific models. In their subsequent study Govindarajan et al. [18] simulated a virtual stress test by progressively increasing left ventricular contraction. They showed that the non‐linear relationship between transvalvular pressure gradient and flow could be attributed to non‐linear changes in valve area during stress, thereby providing an explanation for the heterogeneous pressure–flow behaviour observed among patients [4].

Despite these advances in modelling aortic valves in FSI simulations, verification remains a critical step before FSI simulations can be integrated into the clinical workflow. Simulations can be verified by comparison with data from in vitro experiments. Sigüenza et al. [19] addressed verification by replicating in vitro experiments in their FSI model. Their experiments included a pulse duplicator generating pulsatile flow through a rigid aortic root model with flexible polymer leaflets. High‐speed recordings enabled detailed analysis of valve kinematics, while image‐based velocity measurements provided insights into the flow dynamics. Their results demonstrated strong agreement between simulations and experiments in both leaflet motion and fluid dynamics. However, like earlier studies, their model was based on an idealised, healthy valve geometry.

To the best of our knowledge, no studies have reported experimental verification of patient‐specific FSI models for aortic stenosis. Therefore, the aim of this study is to experimentally verify an aortic stenosis FSI model, using a patient‐specific geometry, including calcifications, and under varying flow conditions from rest to exercise. Verification is performed by comparing the model's haemodynamics and valve dynamics with results from an in vitro set up. This verification is intended to demonstrate the model's potential as a complementary, non‐invasive tool for the assessment of AS severity. Note that we use the term ‘verification’ rather than ‘validation’, since we do not compare the model to the real world, that is, the in vivo situation. Such comparison is required for ‘validation’ according to the definitions by the US Department of Defense [20] and Schlesinger et al. [21], that we adhere. For a more elaborate discussion on the terminology of verification and validation we refer to Oberkampf et al. [22].

To achieve this goal, in vitro experiments were conducted using a mock‐loop circulatory system equipped with patient‐specific silicone rubber valve models, both with and without calcium deposits. The experiments were conducted under varying flow conditions and were replicated in FSI simulations. To verify the FSI model, simulated and experimental results were compared.

## 2 Methods

**FIGURE 1:**
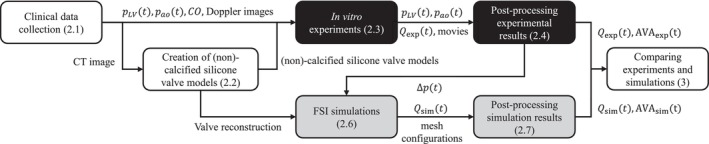
Flowchart summarising steps of this study with black and grey blocks indicating experimental and simulation steps, respectively. Patient data is collected in the clinic, and a CT‐based silicone valve model is created. The experimental setup uses left ventricular, and aortic pressure signals ($p_{\mathrm{LV}}\left(t\right)$, $p_{\mathrm{ao}}\left(t\right)$), cardiac output (CO), Doppler images, and the valve model. Post‐processing yields flow ($Q_{\exp}$) and AVA_exp_ over time. CT‐based valve reconstruction and experimental pressure difference (Δp) are used as input for the simulations. Simulated flow ($Q_{\mathrm{sim}}$) and AVA signals (AVA_sim_) are compared with the experimental values for verification of the simulations. Section references are shown in each block.

This study consisted of several steps, as outlined in the flowchart in Figure 1. The process began with the collection of clinical patient data, described in Section 2.1. The collected clinical data were used in both the in vitro experiments and the FSI simulations. Using the patient‐specific computed tomography (CT) images, a silicone valve model was created (Section 2.2). This silicone valve model, along with additional clinical measurements were mounted into the experimental set up (Section 2.3). The post‐processing of experimental data is detailed in Section 2.4. The numerical method is described in Section 2.5, followed by the development of the FSI model, which aimed to replicate the mock‐loop experiment as accurately as possible (Section 2.6). After post‐processing the simulation results (Section 2.7), the FSI simulations were verified by comparing the simulated results with the corresponding experimental results (Section 3).

### 2.1 Clinical Data Collection

For both the experiments and the numerical simulations, data from a single patient of the cohort of Johnson et al. [4] were used. The patient had a severe aortic stenosis and underwent TAVI. Prior to the procedure, a computed tomography (CT) scan of the aortic valve in closed configuration was performed. Just before TAVI, invasive pressure measurements using two coronary pressure wires (Aeris PressureWire, St. Jude Medical), one in the left ventricle and one in the high ascending aorta, were performed. During the measurements, a step‐wise dobutamine infusion began, with each phase lasting 3 to 5 min. Different dobutamine doses were administered: 0 (baseline), 5, 10, 20 μg/kg/min, from now on abbreviated with d0, d5, d10, d20, respectively. Simultaneously cardiac output (CO) was measured using a Swan‐Ganz catheter with thermodilution capability, via the pulmonary artery. Moreover, continuous wave Doppler images using transesophageal ultrasound imaging were acquired. Directly after the TAVI procedure, the invasive pressure measurement under step‐wise dobutamine infusion was repeated.

### 2.2 Silicone Valve Model

The valve reconstruction from the CT scan was used to create the patient‐specific silicone valve models for the mock‐loop circulation experiments, following the same procedure as Zelis et al. [23]. Two silicone valve models were created: one with calcifications and one without calcifications. The CT scan was imported into Mimics (v18.0, Materialise, Belgium), where the left ventricular outflow tract (LVOT), valve leaflets, calcifications, and aortic root were manually reconstructed. From these reconstructions, a 3D surface mesh was generated, smoothed, and patched to fill any remaining gaps. Using SolidWorks (Dassault Systèmes, France), moulds were designed based on the 3D geometries. The moulds were composed of the inside mould and the outside mould. The inside mould was based on the 3D surface mesh. The outside mould was a cylinder with a diameter of 60 mm. Both moulds were printed in hard resin (VeroWhite, Sculpteo, France). Valve models without calcifications were created by injecting an elastic silicone rubber (Ecoflex 5, Smooth‐On, USA) into the resin moulds. For calcified valve models, calcifications were cast separately in a rubber‐like material (TangoBlack, Stratasys, Israel) to facilitate removal. These moulds were filled with fine grit plaster to replicate calcified structures. The patient‐specific calcifications were then placed onto the inner moulds before silicone rubber was injected to form the complete valve. Lastly, to mimic physiological leaflet separation, the leaflets were manually separated at the coaptation zones based on ultrasound (US) images of the aortic valve in short‐axis view during valve opening and closing.

### 2.3 In Vitro Experiments

#### 2.3.1 Experimental Setup

**FIGURE 2:**
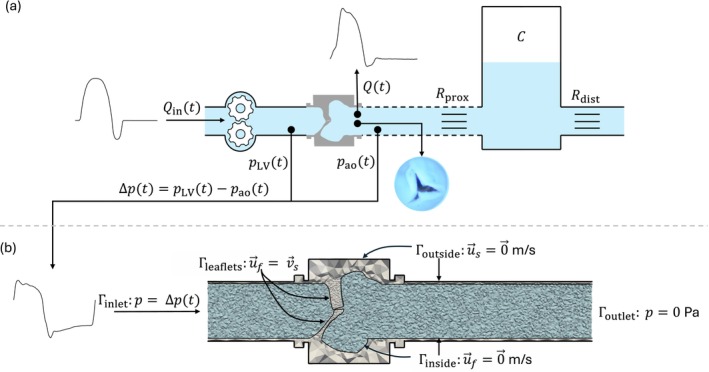
(a) Experimental set up: Flow ($Q_{\text{in}}$) is driven by a gear pump through a silicone valve model. Pressures ($p_{\mathrm{LV}}$) and ($p_{\mathrm{ao}}$) are measured with catheters, and flow ($Q\left(t\right)$) is recorded downstream. Valve motion is recorded with an endoscope. The valve connects to a Windkessel model with proximal and distal resistances, ($R_{\text{prox}}$ and $R_{\text{dist}}$), and capacitance (C) via a flexible tube (dashed line). (b) Simulation set up: The measured pressure drop is prescribed at the inlet ($\Gamma_{\text{inlet}}:p=\Delta p\left(t\right)$), outlet pressure is set to $\Gamma_{\text{outlet}}≔0$ Pa. At the inside of the tube the fluid velocity is set to $\Gamma_{\text{inside}}:{\vec{u}}_{f}$ = $\vec{0}$ m/s, and at the leaflets it is set to $\Gamma_{\text{leaflets}}:{\vec{u}}_{f}={\vec{v}}_{s}$. All out side solid nodes are fixed in all directions ($\Gamma_{\text{outside}}:{\vec{u}}_{s}$ = $\vec{0}$ m/s).

Figure 2a shows a schematic overview of the experimental set up, which is a simplified version of the mock‐loop circulation described by Geven et al. [24]. Flow was generated using a servomotor‐driven gear pump (Liquiflo 37F), which is operated using LabVIEW software (v2014, National Instruments, USA). The silicone valve was placed in a rigid valve housing that was connected to the gear pump with a rigid tube. A flexible tube made of silicone rubber (Young's modulus of 3 MPa and Poisson's ratio 0.5) representing the aorta was attached to the other side of the valve. Lastly, the aorta was connected to a Windkessel model with variable proximal and distal resistances, which allowed for manual tuning of the aortic pressure waveform. Pressures were recorded proximal and distal to the valve using P10EZ pressure sensors (Beckton Dickinson, Sint‐Niklaas, Belgium), and flow is recorded using an ultrasonic flow probe (25 MPXL, Transonic, Maastricht, The Netherlands), with a measurement uncertainty of 10% [25]. Finally, an endoscope (Olympus, Tokyo, Japan) connected to a camera (Nikon, Tokyo, Japan) was placed inside the aorta via the proximal resistance to record images and videos of valve behaviour during the experiments.

#### 2.3.2 Measurement Approach

**FIGURE 3:**
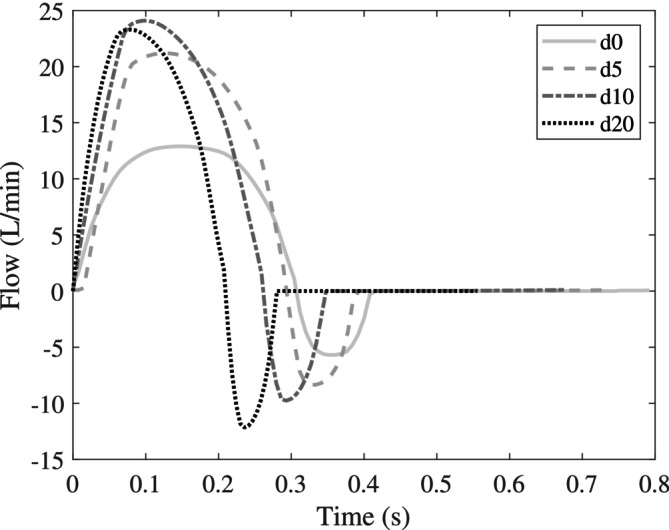
Flow signals used as input for the gear pump in the in vitro experiments for four different dobutamine infusion rates d0, d5, d10 and d20. Shape and duration are reconstructed from Doppler images, and the curves are scaled to match the measured cardiac outputs.

For both the non‐calcified and calcified silicone valve models, four in vitro experiments were performed, each corresponding to a different dobutamine infusion rate (d0, d5, d10 and d20). This resulted in a total of eight experiments. Flow profiles were prescribed, with waveform shape and duration reconstructed from the Doppler images, and scaled to match the cardiac output measured via thermodilution. Since the segment connecting the gear pump was slightly compliant, a negative flow rate with an integral of 5 mL was prescribed to the gear pump to restore realistic proximal pressures. The heart rates corresponding to d0, d5, d10 and d20 were 75, 81, 88 and 107 bpm, and the flow curves were scaled to match cardiac outputs: 3.2, 5.1, 5.5 and 5.1 L/min, respectively. The resulting flow curves prescribed to the gear pump for the four experiments are shown in Figure 3. These curves were used for the experiments with non‐calcified as well as for the calcified silicone valve models.

### 2.4 Post‐Processing of Experimental Results

The pressure signals collected during the experiments were processed to serve as boundary conditions for the simulations (see Section 2.6). For each experiment, we extracted all cycles from the point the signal became periodic until the end of the experiment, for both the LV and aortic pressures. The pressure waveforms were then averaged across cycles to obtain a cycle‐averaged waveform. The cycle‐averaged pressure difference $\Delta p\left(t\right)$ was obtained by subtracting the aortic from the LV pressure waveform. Averaging across all cycles reduced signal noise, resulting in smooth pressure waveforms. The final waveform was then repeated periodically to create a signal spanning six cardiac cycles. To verify the simulations, we used the flow measurements obtained from the experiments. The same method used for the pressures was applied to derive the cycle‐averaged flow and its standard deviation. The mean transvalvular flow ($Q_{\text{mean}}$), that is, the mean flow during the ejection period, was obtained for each cycle. It was calculated by integrating the flow curve $Q\left(t\right)$ between the crossing point where $p_{\mathrm{LV}}>p_{\mathrm{ao}}$ and where $Q\left(t\right)<0$, and dividing by the duration of this interval. Likewise, the mean transvalvular pressure drop ($\Delta p_{\text{mean}}$) was computed over the period where the $\Delta p\left(t\right)$ > 0 [4]. For each experiment, the average and standard deviation of $Q_{\text{mean}}$ and $\Delta p_{\text{mean}}$ over the cycles were determined.

**FIGURE 4:**
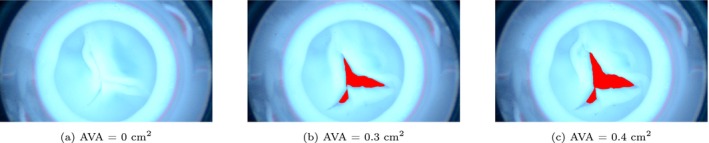
Three snapshots of the endoscope movie during the experiment with AVA annotated in red.

The videos recorded by the endoscope were loaded into MATLAB 2021a. AVA was estimated from each frame. First the images were converted to greyscale, and inverted, so that the valve opening appeared with higher intensity than the surrounding regions. Then, a fixed intensity threshold was applied to create a binary mask highlighting the valve opening. Morphological operations were subsequently performed on the binary mask, including hole filling within objects, removal of small isolated regions, and closing of small gaps between connected objects. To isolate the region of interest, a circular mask, defined by the valve's centre and radius, was applied to remove the background. The connected parts of the binary images were analysed based on area and the two largest regions were assumed to correspond to the valve opening. The area in number of pixels was then converted to an area in cm^2^. These steps were done for all frames to obtain an AVA signal as a function of time. Figure 4 shows three different snapshots of an experimental movie with the AVA annotated. It was assumed that the chosen threshold to create the mask was the most important source of uncertainty in the AVA determination. Therefore, AVAs were also determined using 110% and 90% of the fixed threshold, yielding a lower and an upper limit for AVA, respectively.

### 2.5 Numerical Methods

**FIGURE 5:**
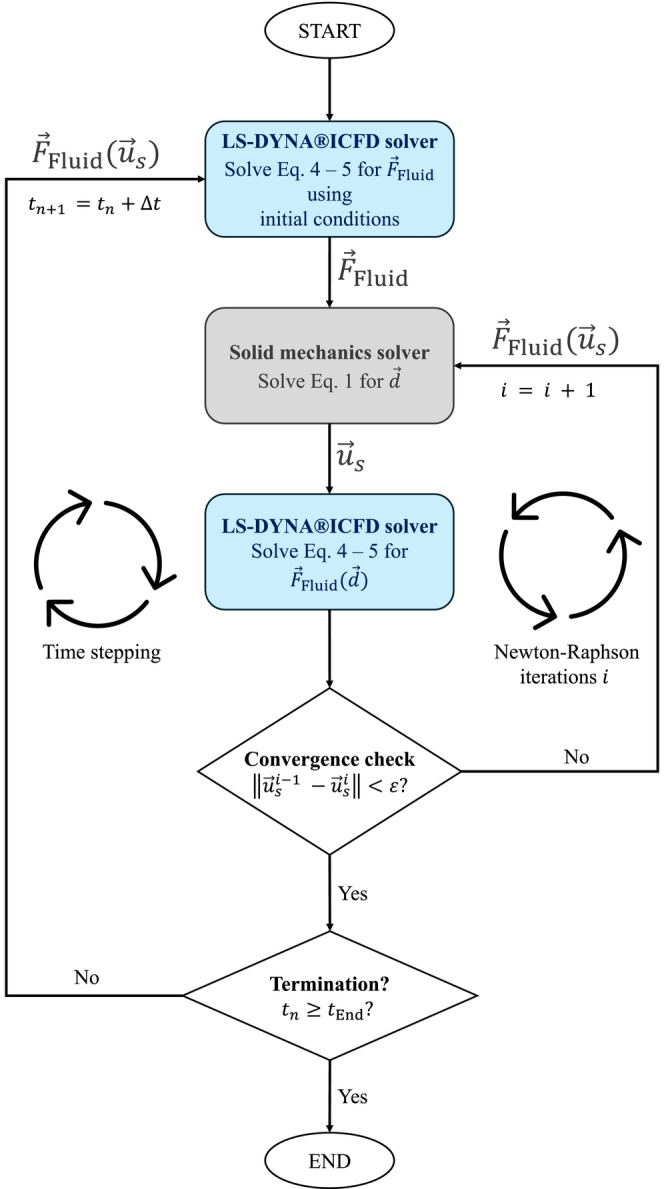
Flowchart of the strong FSI coupling framework. Fluid and solid sub‐problems are solved in Newton–Raphson iterations i at each time step. The LS‐DYNAICFD solver transfers fluid forces ${\vec{F}}_{\text{Fluid}}$ to the solid mechanics solver that computes the resulting displacements ${\vec{u}}_{s}$. Convergence is reached when the difference in ${\vec{u}}_{s}$ between iterations falls below a threshold ε. The algorithm proceeds to the next time step $t_{n+1}$.

The FSI model was built in the commercial finite element solver ANSYS LS‐DYNA R16.0 (Ansys Inc., Pittsburg PA, United States of America). A partitioned approach was adopted, where the fluid and solid sub‐problems are solved independently using the incompressible computational fluid dynamics (LS‐DYNAICFD) solver and the solid mechanics solver, respectively. The coupling between the two solvers is achieved by exchanging interface forces and displacements to account for the interaction between fluid and structure. A schematic overview of the algorithm employed in LS‐DYNA is visualised in Figure 5.

#### 2.5.1 Fluid Problem

The fluid was modelled as an incompressible Newtonian fluid. The governing equations for the fluid solver consist of the incompressible Navier–Stokes equations:

(1) $$\nabla \cdot {\vec{u}}_{f}=0,$$

(2) $$\rho_{f}\left(\frac{\partial {\vec{u}}_{f}}{\partial t}+\left(\left({\vec{u}}_{f}-{\vec{u}}_{m}\right)\cdot \nabla \right){\vec{u}}_{f}\right)=-\nabla p+\mu \nabla^{2}{\vec{u}}_{f}+\rho_{f}{\vec{f}}_{f},$$

with ${\vec{u}}_{f}$, the fluid velocity vector field, ${\vec{u}}_{m}$, the velocity of the moving mesh, t the time, $\rho_{f}$, the fluid density, p, the pressure, μ, the dynamic viscosity and ${\vec{f}}_{f}$, the external forces per unit of mass applied to the fluid. The boundaries of the fluid domain are not fixed as a result of the movement of the structure. To deal with this mesh movement, the Arbitrary Lagrange Euler (ALE) [26] approach is used (Equation 2), together with the default mesh movement algorithm that solves a compressible linear elasticity problem to propagate the velocity of the moving fluid–structure interface to the interior of the fluid domain. A backward differences of second‐order implicit scheme was applied for time integration. To solve Equations (1 and 2) in a computationally efficient way, the pressure unknowns are segregated from the velocity unknowns using the fractional step method, involving three sub‐steps within each time step n. The first step involves computing a predictor velocity ${\vec{u}}^{*}$ by solving

(3) $${\vec{u}}^{*}-\Delta t\;\nu \nabla^{2}{\vec{u}}^{*}+\Delta t\left(\left({\vec{u}}^{L}-{\vec{u}}_{m}\right)\cdot \nabla {\vec{u}}^{*}\right)=2{\vec{u}}^{n}-\frac{1}{2}{\vec{u}}^{n-1}-\frac{\Delta t}{\rho }\nabla p^{n}+\Delta t\;{\vec{f}}^{n+1},$$

with ν the kinematic viscosity of the fluid. Note that the subscripts f for fluid are omitted for readability. To linearise the convection term, the first velocity is estimated by ${\vec{u}}^{L}=2{\vec{u}}^{n}-{\vec{u}}^{n-1}$. The predicted velocity ${\vec{u}}^{*}$ does not yet satisfy the incompressibility constraint (Equation 1), and is typically not divergence‐free. To correct for the divergence in the predictor velocity, a Poisson equation for the pressure field $p^{n+1}$ is derived from the incompressibility condition (Equation 1),

(4) $$\nabla^{2}\left(p^{n+1}-p^{n}\right)=\frac{\rho }{\Delta t}\left(\nabla \cdot {\vec{u}}^{*}\right).$$

This equation determines the pressure necessary to adjust the velocity field so that it becomes divergence‐free. The equation is iteratively solved with a maximum of 10 sub‐iterations, by iterating between pressure ($p^{n+1}$) and velocity (${\vec{u}}^{*}$). In the final step, the predictor velocity ${\vec{u}}^{*}$ is corrected using the pressure computed in the previous step, resulting in the final velocity ${\vec{u}}^{n+1}$

(5) $$\frac{3}{2}{\vec{u}}^{n+1}={\vec{u}}^{*}+\frac{\Delta t}{\rho }\nabla \left(-p^{n+1}+p^{n}\right).$$

The weak formulations of Equations ((3), (4), (5)) are discretised in space with the Finite Element Method (FEM), using linear tetrahedral elements and solved in the LS‐DYNAICFD solver. Solving Equations ((3), (4), (5)) is very prone to stability issues. Therefore, pressure and convection stabilisation is applied using orthogonal subscale stabilisation for which we refer to the work of Codina [27] for a detailed description of this stabilisation technique.

#### 2.5.2 Solid Problem

For the solid domain, the momentum balance equation was solved in a Lagrangian formulation.

(6) $$\nabla \cdot \sigma_{s}+\rho_{s}{\vec{f}}_{s}=\rho_{s}\frac{d^{2}{\vec{u}}_{s}}{{\mathrm{dt}}^{2}},$$

with $\rho_{s}$, the density of the solid, ${\vec{u}}_{s}$ the displacement of the material points, $\sigma_{s}$, the Cauchy stress tensor, and ${\vec{f}}_{s}$, the body forces per unit mass, acting on the solid. Equation (6) was discretised in space with FEM, using linear tetrahedral elements and solved using an implicit Newmark time integration scheme with γ = 0.6, and β=0.4.

#### 2.5.3 Coupling

The solid and fluid solvers are coupled using a strongly coupled fluid–structure interaction (FSI) scheme, in which the fluid and solid sub‐problems are solved iteratively at each time step until convergence is achieved. A flowchart of the FSI algorithm implemented in LS‐DYNA is shown in Figure 5. The algorithm starts with a fluid solve by the LS‐DYNAICFD solver, that solves Equations ((3), (4), (5)) using the initial fluid conditions. The resulting hydrodynamic forces (${\vec{F}}_{\text{Fluid}}$) are transferred to the solid mechanics solver, that solves Equation (6), yielding the structural displacements (${\vec{u}}_{s}$). Using these displacements and the material constitutive model, the internal solid forces ${\vec{F}}_{\text{Solid}}$ are calculated. Subsequently, the displacements are passed back to the LS‐DYNAICFD solver, which recalculates the fluid forces while accounting for the deformation of the fluid domain using the ALE framework [26]. Re‐meshing operations on the tetrahedron elements were performed when needed, while keeping the triangular surface mesh of the leaflets. These steps are repeated within Newton–Raphson iterations, indexed by i, until convergence is reached. Convergence is achieved when the normalised change in ${\vec{u}}_{s}$ between successive iterations falls below a predefined threshold ε. Then, the algorithm advances to the next time step. To enhance stability and prevent divergence within the Newton–Raphson loop, LS‐DYNA incorporates line search iterations (not shown in Figure 5). During these iterations a scalar parameter $s\in \left[0,1\right]$ is determined to minimise the out‐of‐balance force (${\vec{F}}_{\text{Fluid}}$—${\vec{F}}_{\text{Solid}}$), by iteratively updating

(7) $${\vec{u}}_{s}^{i}={\vec{u}}_{s}^{i-1}+s\Delta {\vec{u}}_{s}.$$

When the optimal s is found, the algorithm proceeds to the next Newton–Raphson iteration.

Since the density of the fluid and the solid are very similar in this study, the ‘added‐mass‐effect’ is significant. The added mass is the inertia added to the system, because a moving solid also has to move volume of the surrounding fluid, as they cannot occupy the same space simultaneously. This effect can lead to stability issues. To overcome these issues, the contribution of the solid elements on the interface is added to the pressure Laplace matrix, which is part of the discretised form of Equation (4). For a detailed description of this method we refer to Idelsohn et al. [28]. Moreover, in FSI simulations, LS‐DYNA introduces ‘artificial compressibility’ of the fluid, near the FSI interface, meaning that the solver may relax the incompressibility constraint locally to facilitate convergence.

### 2.6 FSI Simulations

#### 2.6.1 Simulation Setup—Fluid

**FIGURE 6:**
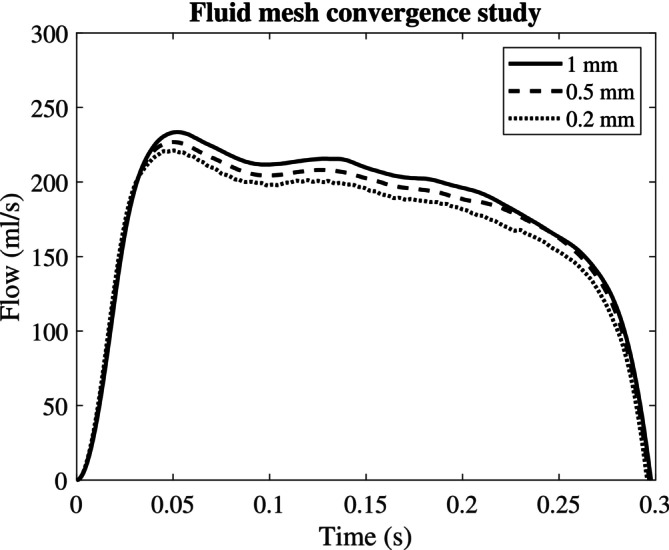
Fluid mesh convergence study: Flow as a function of time for a CFD simulation at the peak systolic configuration of the valves for three different fluid volume mesh resolutions.

To create the fluid domain, the inside of the tube is extruded by 4 and 9 cm proximally and distally to the valve, respectively, and capped with an inlet and outlet surface. A mesh convergence study was conducted for the fluid problem by performing a CFD simulation in the peak systole configuration of the valves. The pressure difference signal obtained from experiment d0 and a constant zero pressure were prescribed at the inflow and outflow boundaries, respectively. Eight cardiac cycles were simulated using three different meshes with volume element edge sizes of 1.0, 0.5 and 0.2 mm. The resulting flow curves are visualised in Figure 6.

**FIGURE 7:**
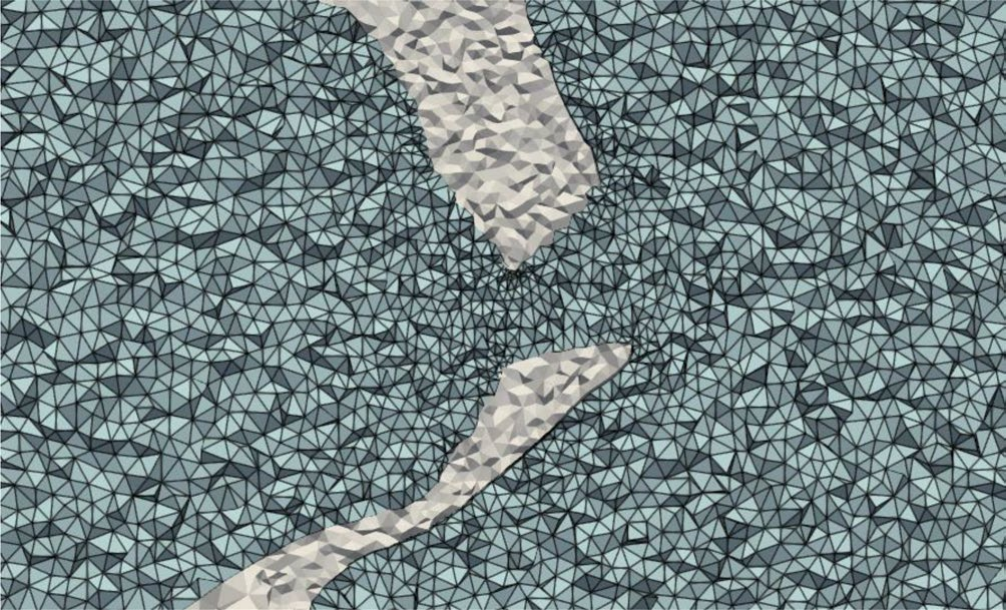
Fluid mesh in peak systole, zoomed in on valve leaflets.

The peak flows equalled 232, 226 and 221 mL/s, the mean transvalvular flows, 172, 169 and 170 mL/s, and the stroke volumes 51, 50 and 50 mL for the 1, 0.5 and 0.2 mm mesh resolutions, respectively. The last mesh refinement resulted in only a 2%, 0.5% and 0.1% difference in peak flow, mean flow and stroke volume, compared to the previous mesh, while computational time drastically increased from 20 min to 13 h to simulate only one systolic phase. Given this trade‐off, the element edge size of 0.5 mm was chosen, yielding 295,131 tetrahedral elements in the fluid domain. Meshing was performed using the automatic mesher in the LS‐DYNAICFD solver. Re‐meshing was triggered when inverted elements were detected or element quality deteriorated. The fluid mesh is depicted in blue in Figure 2b and Figure 7.

The viscosity and density of the fluid were set to those of water, since water was also used in the experimental setup. Using the cycle‐averaged measured pressures in the experiment (Sections 2.3 and 2.4), the pressure difference over time ($\Delta p\left(t\right)=p_{\mathrm{LV}}\left(t\right)-p_{\mathrm{ao}}\left(t\right)$) was calculated. Since we were interested in the pressure drop across the valve, rather than absolute pressures, the pressure difference Δp (Equation 8) and a constant pressure of 0 Pa (Equation 9) were prescribed as Dirichlet boundary conditions at the inflow and outflow boundaries of the fluid domain, respectively. A no‐slip condition was used at the fluid domain's other boundaries (Equation 10), where the boundaries overlapping the leaflets have a velocity according to the displacements calculated by the solid solver (Equation 11). Moreover, two Neumann boundary conditions were applied. The first one assumes fully developed flow at the outflow boundary (Equation 12). Also, a boundary condition is needed for the Poisson's equation (Equation 4). Therefore, the pressure gradient over all boundaries, except for the outflow boundary were assumed to be 0 Pa (Equation 13). In summary, the fluid boundary conditions with the corresponding equations were:

(8) $$p^{n+1}=\Delta p\left(t\right)\;\mathrm{on}\;\Gamma_{\text{inflow}}\;\text{in Equation}\;4,$$

(9) $$p^{n+1}=0\;\mathrm{Pa}\;\mathrm{on}\;\Gamma_{\text{outflow}}\;\text{in Equation}\;4,$$

(10) $${\vec{u}}^{*}=\vec{0}\;m/s\;\mathrm{on}\;\Gamma_{\text{inside}}\;\text{in Equation}\;3,$$

(11) $${\vec{u}}^{*}={\vec{v}}_{s}\;\mathrm{on}\;\Gamma_{\text{leaflets}}\;\text{in Equation}\;3,$$

(12) $$\nabla u_{k}^{*}\cdot \vec{n}=0\;\mathrm{Pa}\;\mathrm{on}\;\Gamma_{\text{outflow}}\;\text{in Equation}\;3,$$

(13) $$\nabla p^{n+1}\cdot \vec{n}=0\;\mathrm{Pa}\;\mathrm{on}\;\Gamma \notin \Gamma_{\text{outflow}}\;\text{in Equation}\;4,$$

with subscript k referring to dimensions, x,y, and z, and ${\vec{v}}_{s}$ the velocity of the solid. LS‐DYNA's backflow stabilisation method, that uses velocity time derivatives to suppress rapid, unphysical velocity changes at the boundaries, was employed.

Since the Reynolds number in the simulation exceeds 2500, the flow is considered to be in the turbulent regime. Resolving all turbulence scales through direct numerical simulation (DNS) would require an impractically fine mesh and high computational resources. On the other hand, Reynolds‐Averaged Navier–Stokes (RANS) approaches, which model all turbulence scales, are unsuitable for this application, as they tend to wash out unsteady vortices that are important to accurately capture valve dynamics. To balance accuracy and computational efficiency, we used the Large Eddy Simulation (LES) approach. LES resolves the large, energy‐containing eddies while modelling the smaller scales using the Smagorinsky sub‐grid scale model [29]. This method has also been successfully applied in previous studies on simulating cardiovascular flows [19, 30]. As in Sigüenza et al. [19], we evaluated the Pope criterion which estimates the ratio of sub‐grid scale turbulent kinetic energy $k_{\mathrm{SGS}}$, lying below the LES filter width Δ, to the total turbulent kinetic energy k [19, 31]:

(14) $$\frac{k_{\mathrm{SGS}}}{k}=\frac{3}{2}C{\left(\frac{\Delta }{\mathrm{\pi L}}\right)}^{\frac{2}{3}},$$

where Pope's constant C = 1.5, Δ = 0.5 mm, (mesh resolution), and L = 13 mm (characteristic length scale, corresponding to the tube radius). This yields $k_{\mathrm{SGS}}/k$ = 12%, meaning that 88% of the turbulent kinetic energy is fully resolved. This satisfies the Pope criterion, which states that a good LES should resolve at least 80% of the kinetic energy [30, 31], thereby justifying the chosen mesh resolution of 0.5 mm.

#### 2.6.2 Simulation Setup—Solid

For the solid domain, the inner surface of the 3D reconstruction from CT (Section 2.2) was cut out of a tube geometry with the same dimensions as the outer mould in Section 2.2 using SolidWorks (Dassault Systèmes, France), which resulted in a surface mesh representing the boundaries of the solid domain. To create three separate leaflets, the leaflet edges were traced on both the LV and aortic sides of the valve. Based on these tracings, a propeller‐shaped solid of thickness 2 mm was removed from the geometry, leading to the three leaflets, with 2 mm in between.

The inside of the surface was meshed with tetrahedral elements in ANSYS Workbench Meshing R23.1 (ANSYS Inc., United States). To avoid volumetric locking, an artificial stiffening of elements that often occurs when modelling (nearly) incompressible materials, pressure‐averaged tetrahedral elements were employed [32]. These elements mitigate volumetric locking by averaging the volumetric strain over neighbouring elements, resulting in a smoother and more stable pressure response. The leaflet elements had an edge size of 0.5 mm, equal to the edge size of the fluid elements at the fluid–structure interface. The remaining parts had edge sizes ranging between 1 and 10 mm, yielding a total number of elements of 264,710. The solid mesh is depicted in grey in Figure 2b.

The outside mesh points were fixed in all directions for the solid domain, so they could not move.

(15) $$\Gamma_{\text{outside}}:{\vec{u}}_{s}=\vec{0}\;m/s.$$

Only the leaflets were allowed to move, according to an applied pressure exerted by the fluid.

The leaflet material was modelled as nearly incompressible using a Neo‐Hookean material model with a mass density of 1000 kg/m^3^, a Poisson's ratio of 0.4999 and shear modulus G. Since the exact shear modulus G of the silicone valve models was unknown, it was calibrated by matching simulated stroke volumes to experimental measurements under baseline conditions (d0) with the non‐calcified valve. This calibration process, detailed in Appendix A, yielded a shear modulus of 79 kPa. Using this shear modulus, simulations were subsequently performed for dobutamine infusion rates corresponding to d5, d10, and d20. To verify that the calibration was not dependent on the underlying data, the shear modulus was additionally calibrated on experiment d5, yielding a shear modulus of 75 kPa (Appendix A).

For the simulations including calcifications, CT‐based reconstructions of the calcified deposits were used to identify the corresponding leaflet mesh elements. These elements were assigned a higher shear modulus, while the surrounding non‐calcified elements retained the calibrated value of 79 kPa. To calibrate the shear modulus for the calcifications, simulated stroke volumes were matched to the experimental values under baseline conditions (d0) with the calcified silicone valve, yielding a shear modulus of 10 MPa (Appendix A). The use of volume‐averaged tetrahedral elements required uniform compression moduli across adjacent elements. Therefore, to compensate for the higher shear modulus of the calcifications, the Poisson's ratio had to be slightly decreased to 0.4925. This adjustment ensured that the compression moduli of the calcifications and surrounding leaflet tissue matched and prevented numerical instabilities. The density for the calcifications was assumed to be the same as that of the surrounding leaflets. This is a valid assumption, since in the experiment the density of both calcifications and the silicone rubber is similar as well.

Lastly, to prevent the leaflets from intersecting, a segment‐to‐segment contact algorithm based on the mortar method is used [33], with a contact thickness of 0.1 mm, a contact stiffness scaling factor of 0.005, and a maximum penetration depth of 0.06 mm.

#### 2.6.3 Simulation Approach

**FIGURE 8:**
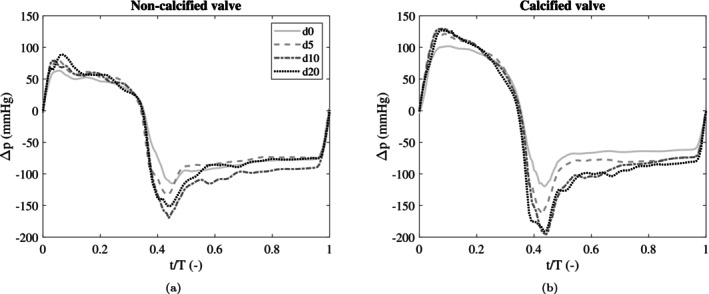
Pressure difference curves measured in the experiments, and prescribed at the inflow boundary of the FSI model, for four dobutamine infusion levels, for the non‐calcified valve (a) and the calcified valve (b). Pressure is plotted as a function of time, relative to the cycle time (T), which is 0.8, 0.75, 0.7 and 0.57 s for d0, d5, d10 and d20, respectively.

In total, eight FSI simulations were conducted, replicating the eight in vitro experiments (Section 2.3). In each FSI simulation, six complete cardiac cycles were simulated with a fixed time step size of 0.0002 s. Simulations were performed on the Snellius supercomputer (AMD EPYC 7H12 64‐Core Processor) on 32 cores. The periodic, experimental pressure difference signals ($\Delta p\left(t\right)$) with six cardiac cycles (Section 2.4) were prescribed at the inflow boundary of the fluid domain. For the simulations with dobutamine infusion rates d0, d5, d10 and d20, the mean transvalvular pressure drops were 44, 52, 51 53 mmHg, respectively for the non‐calcified valve. For the simulations with the calcified valve, the mean transvalvular pressure drops equalled 77, 89, 87 and 85 mmHg, respectively. The corresponding, pressure difference curves for the non‐calcified are shown in Figure 8a and for the calcified valve in Figure 8b. The cycle times for d0, d5, d10 and d20 equalled 0.8, 0.75, 0.7 and 0.57 s, respectively. Cross‐validation simulations were performed on the non‐calcified valve, by using the shear modulus calibrated on d5 for dobutamine infusion rates d0, d10 and d20.

### 2.7 Post‐Processing Simulation Results

The flows and pressures were automatically computed and saved in LS‐DYNA at the inlet and the outlet of the fluid domain. The mean transvalvular flows ($Q_{\text{mean}}$) were computed for each of the six cycles in the same way as described for the experimental results (Section 2.4). Subsequently, the average and inter‐cycle standard deviation were calculated. Extracting the AVA from the simulations using the same method as for the experiments was challenging, as the mesh configurations from the simulations were in 3D, while the experimental images were in 2D. To enable a fair comparison between experimental and simulated AVAs, the 3D point cloud representing the mesh was projected onto a 2D plane. A 2D grid was then defined with a resolution of 150 × 150, where grid points overlapping with projected mesh points were assigned a value of 1, and all others a value of 0, resulting in a binary image. Holes in the binary image were filled. Subtracting the original binary image from the filled one and extracting the largest connected component yielded the AVA in pixels, which was then converted to cm^2^. Here, we assumed the chosen resolution of the 2D grid was the most important source of uncertainty. Therefore, we also calculated the AVAs with a resolution of 110%, and 90% of the default resolution, yielding a lower and an upper limit for AVA, respectively.

## 3 Results

In vitro experiments were performed for the four dobutamine infusion rates d0, d5, d10 and d20, using the non‐calcified and calcified silicone valve in the mock‐loop circulation. These eight experiments were replicated in the FSI model. To verify the FSI model, the simulation results are compared to the experimental results by taking into account both flow dynamics (Section 3.1) and valve dynamics (Section 3.2). All results are summarised in Table 1.

**TABLE 1:** Summary of results for all experiments and simulations.

	Dobutamine infusion rate (μg/kg/min)	Cycle time (s)	Mean transvalvular Δ*p* (mmHg)	Mean transvalvular flow (mL/s) (mean ± uncertainty)		AVA (cm^2^) (range)	
				Experiment	Simulation	Experiment	Simulation
Non‐calcified	0	0.80	44	143 ± 14	149 ± 2	0.40 (0.39–0.44)	0.44 (0.43–0.45)
	5	0.76	52	219 ± 22	222 ± 5	0.67 (0.63–0.71)	0.69 (0.68–0.69)
	10	0.70	51	228 ± 23	209 ± 4	0.78 (0.73–0.82)	0.63 (0.62–0.64)
	20	0.57	53	215 ± 22	228 ± 4	0.73 (0.68–0.77)	0.77 (0.76–0.77)
Calcified	0	0.80	77	137 ± 14	154 ± 8	0.39 (0.33–0.44)	0.37 (0.35–0.37)
	5	0.76	89	216 ± 22	205 ± 8	0.51 (0.44–0.58)	0.50 (0.49–0.52)
	10	0.70	87	226 ± 23	208 ± 5	0.48 (0.41–0.54)	0.55 (0.53–0.56)
	20	0.57	85	214 ± 21	201 ± 7	0.57 (0.49–0.64)	0.54 (0.53–0.55)

*Note:* Note that for mean transvalvular flow, the uncertainty in experiments is the measurement uncertainty, while for the simulations it represents the variability between cycles.

### 3.1 Flow Dynamics

**FIGURE 9:**
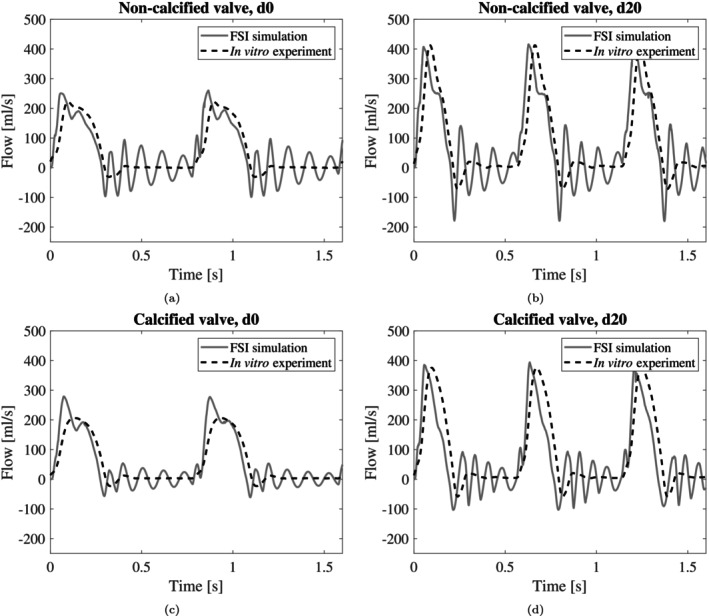
Comparison of simulated and experimental flow signals for baseline d0 (a, c) and highest dobutamine infusion rate d20 (b, d), without calcifications (a, b) and with calcifications (c, d) for the first 1.6 s.

Figure 9 visualises the measured and computed flow waveforms for d0 and d20 for both the non‐calcified and calcified valves. The overall shape of the simulation waveforms agreed quite well with the experimental flows. However, in diastole, the simulations showed an oscillating flow, while the experiments showed a more constant flow. A second notable difference was that the simulated flow increased faster compared to the experimental flow (approximately 4000 vs. 2000 mL/s^2^ for d0 and 8000 vs. 5000 mL/s^2^ for d20), for the non‐calcified valve (Figure 9a,b). For the simulations with the calcified valve (Figure 9c,d), the ejection period was shorter compared to the experiments (0.27 vs. 0.3 s for d0, and 0.19 vs. 0.22 s for d20). For d0, the simulated peak flow was higher, compared to the experimental value (279 mL/s vs. 206 mL/s).

**FIGURE 10:**
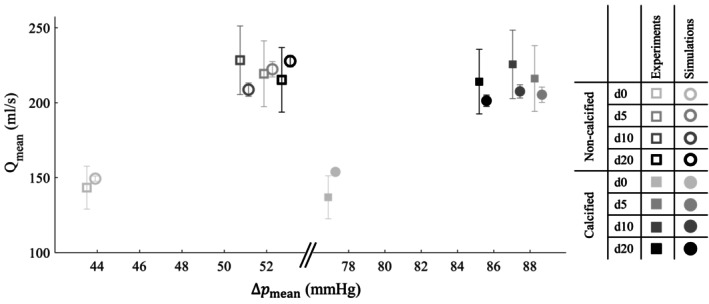
Cycle‐averaged mean transvalvular flow ($Q_{\text{mean}}$), as a function of mean transvalvular pressure drop ($\Delta p_{\text{mean}}$), for all FSI simulations and in vitro experiments, shown for different dobutamine infusion rates and with and without valve calcifications. Error bars indicate inter‐cycle standard deviation for the simulations, and the 10% measurement uncertainty for the experiments. Note that the pressure drop is identical in both the experiments and the simulations, but a slight horizontal off‐set is used to improve visual distinction between the two.

The mean transvalvular flow for all experiments and simulations is shown in Figure 10 and Table 1. Overall, the simulation data points closely aligned with those from the experiments, indicating good agreement. In the simulations with the non‐calcified valve, the expected positive correlation between flow and pressure drop was clearly observed. However, this trend was not evident in the experimental data. Despite this discrepancy, the simulated mean transvalvular flow fell within the measurement uncertainty of 10% of the flow probe used in the experiment [25]. On the contrary, for d0 in the calcified valve, the mean transvalvular flow slightly exceeded the experimental uncertainty range. For dobutamine infusion rates, d5, d10 and d20, the simulated mean transvalvular flows were slightly lower than the experimental values but remained within the experimental uncertainty bounds (Figure 10). Moreover, for both the non‐calcified and calcified valve, the experimental and simulation values for flow and pressure drop fall within the physiological range reported for the same patient in the study by Johnson et al. [4].

**FIGURE 11:**
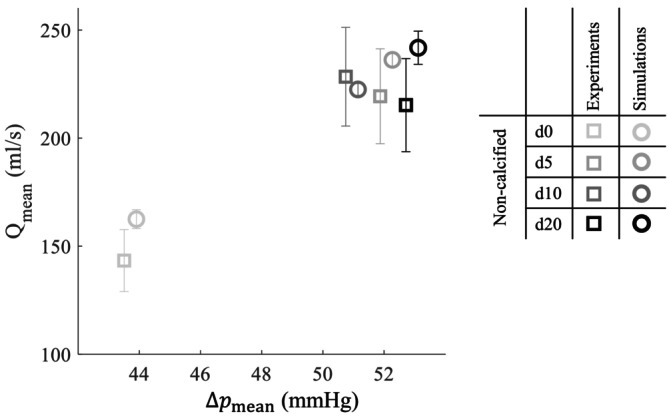
Cycle‐averaged mean transvalvular flow ($Q_{\text{mean}}$), as a function of mean transvalvular pressure drop ($\Delta p_{\text{mean}}$), for all FSI simulations, calibrated on d5 and the in vitro experiments, without valve calcifications. Error bars indicate inter‐cycle standard deviation for the simulations, and the 10% measurement uncertainty for the experiments. Note that the pressure drop is identical in both the experiments and the simulations, but a slight horizontal off‐set is used to improve visual distinction between the two.

To cross‐validate the shear modulus estimation, simulations with the non‐calcified valve were also performed with the shear modulus of 75 kPa, that was the result of the calibration with d5. The resulting mean transvalvular flow as a function of transvalvular pressure drop is shown in Figure 11. For d5 and d10, the simulated flows fell within the experimental uncertainty bounds, whereas for d0 and d20, the flows were slightly overestimated and fell just outside these bounds.

### 3.2 Valve Dynamics

**FIGURE 12:**
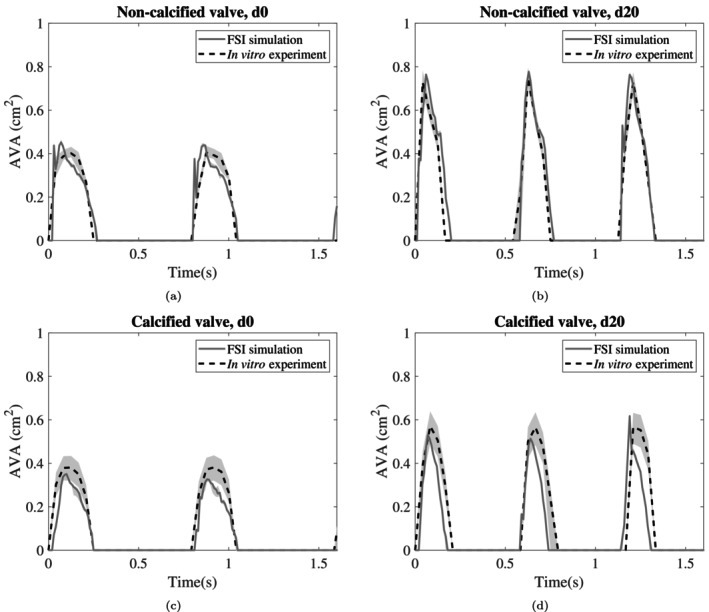
AVA as a function of time for the lowest (d0) (a, c) and the highest (d20) (b, d) dobutamine infusion rate, for both the simulations and the experiments. The light shaded bands indicate the uncertainty of the AVA measurements.

In Figure 12 AVA as a function of time is visualised for the FSI simulations and the in vitro experiments, for d0 and d20, and for both the calcified and non‐calcified valve. The curves will be compared based on four key characteristics of valve dynamics: rate of opening, rate of closing, maximum opening area, and ejection time, that is, the time that the valve remains open. For d0 in the non‐calcified valve (Figure 12a) the simulated valves opened quicker (in 70 ms vs. in 130 ms) than in the experiments, but closed more slowly (in 200 vs. 150 ms). The maximum AVA and ejection time are approximately the same. For d20 (Figure 12b) all four characteristics were approximately equal. Turning to the calcified valve, d0 (Figure 12c) shows similar opening and closing rates between the simulation and experiment. However, the simulations slightly underestimated the maximum AVA (0.35 vs. 0.38 cm^2^). The ejection time was similar. For d20 (Figure 12d), the opening and closing rates were similar, but the maximum AVA varies across cycles. In the first two cycles, the simulation showed a lower AVA than the experiment (0.51 vs. 0.57 cm^2^), but in the third cycle, the simulated AVA exceeded the experimental AVA (0.62 vs. 0.57 cm^2^). Additionally, the simulated ejection time is consistently shorter than in the experiments (0.18 vs. 0.21 s). In summary, the simulations matched the experimental results more closely for the non‐calcified valve. For the calcified valve, however, discrepancies in maximum opening area and ejection time, were observed.

**FIGURE 13:**
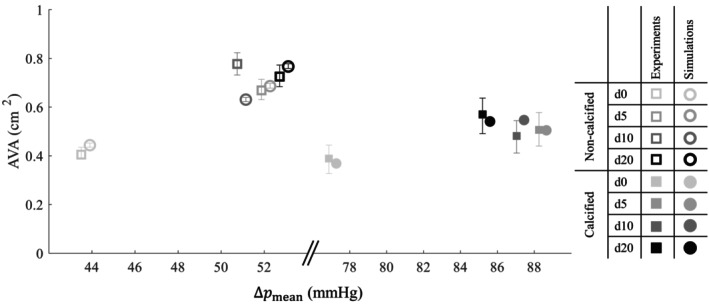
Cycle‐based maximum aortic valve areas (AVAs) in all simulations and experiments, shown for different dobutamine infusion rates and with/without valve calcifications. Error bars indicate the upper and lower limit for AVA, indicating measurement uncertainty. The results were ordered based on increasing transvalvular pressure drop ($\Delta p_{\text{mean}}$).

The maximum AVA values for all experiments and simulations are visualised in Figure 13 and summarised in Table 1. Across all dobutamine infusion rates, except for d10, the experimental and simulated AVA values showed close agreement for the non‐calcified valve. However, the results at d10 deviated from this trend (0.64 ± 0.01 vs. 0.78 ± 0.03 cm^2^). For the calcified valve, the simulated maximum AVA values fell within the experimental measurement uncertainty range.

**FIGURE 14:**
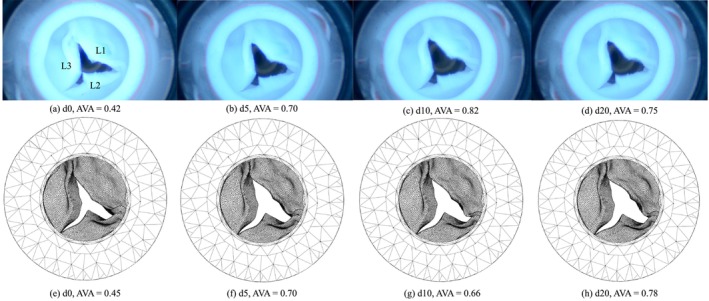
Snapshots of the maximum AVA over all cycles for the non‐calcified valve in the in vitro experiments (a–d), and snapshots of the maximum AVA over all cycles in the FSI simulations (e–h) for dobutamine infusion rates d0, d5, d10 and d20, respectively. The three leaflets are labelled with L1, L2 and L3, annotated in a.

**FIGURE 15:**
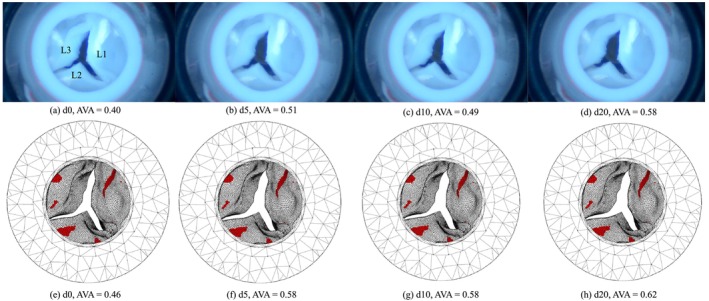
Snapshots of the maximum AVA over all cycles for the calcified valve, in the in vitro experiments (a–d), and snapshots of the maximum AVA over all cycles in the FSI simulations (e–h) for dobutamine infusion rates d0, d5, d10 and d20, respectively. In the simulations, the calcified elements are shown in red. The three leaflets are labelled with L1, L2 and L3, annotated in a.

Figures 14 and 15 present snapshots of both the experiments and simulations at the time of maximum AVA across all cardiac cycles, for the non‐calcified and calcified valve respectively. In addition to the close agreement in AVA values, the shape of the valve opening also shows strong visual similarity between the experiments and simulations. For example, the characteristic bending of the leaflet L3 (Figure 14) was consistently observed in both the experimental and simulated data for all dobutamine infusion rates. Movies of valve movement in the in vitro experiments and the FSI simulations are provided in the Supporting Information for the non‐calcified valve (Movies S1–S4) and the calcified valve (Movies S5–S8).

## 4 Discussion

The aim of this study was to verify a patient‐specific FSI model of aortic stenosis with calcifications, under varying flow conditions, by comparing it to the results of an in vitro mock‐loop experiment.

The mock‐loop experiment was based on imaging data and pressure, and flow measurements in patients with severe AS [4]. The silicone valve models, both with and without calcifications, used in the mock‐loop were based on reconstructions obtained from CT acquisitions. The same patient‐specific valve geometries and calcification patterns were used to set up our FSI simulations. We were able to simulate the valve dynamics and haemodynamics for six full cardiac cycles for different flow conditions, based on patient measurements in rest, and under dobutamine infusion rates of 5, 10 and 20 μg/kg/min. We did simulations with the non‐calcified and calcified valves and compared them to the corresponding mock‐loop experiments.

A good overall agreement was observed between flow waveforms of the simulations and the experiments with the non‐calcified valve. For all four dobutamine infusion rates, mean transvalvular flow $Q_{\text{mean}}$ values of the simulations were close to the values of the experiments, and fell within the measurement uncertainties of ultrasonic flow probe. The mean transvalvular pressure drops and flows observed in the simulations were in the same physiological ranges as reported in Johnson et al. [4], indicating that we were able to simulate physiologically‐plausible haemodynamic conditions and valve dynamics for this patient. The simulated AVA as a function of time matched closely to that of the experiments for the non‐calcified valve, except for d10. For d10, the simulated maximum AVA was significantly lower compared to the experimental value, which was probably due to uncertainties in the pressure measurements. In addition to the overall agreement in AVA values, the shape of the valve opening was very similar, further supporting the ability of our FSI model to capture realistic valve behaviour.

For the calcified valve a good overall agreement between FSI simulations and in vitro experiments was observed as well. However, for d0, the simulated mean transvalvular flow slightly exceeded to experimental uncertainty bounds. This deviation is due to the use of experiment d0 for calibrating the shear modulus. The shorter ejection period in the calcified valve simulation required a higher peak flow to match the experimentally observed stroke volume, resulting in an elevated mean transvalvular flow. For dobutamine infusion rates, d5, d10 and d20, the simulated mean transvalvular flows were slightly lower than the experimental values, again due to the reduced ejection duration. The reduced ejection durations were likely due to uncertainties in the spatial distribution of the calcifications. In the experimental setup, calcifications were manually placed onto the silicone rubber valve moulds. Consequently, their positions did not exactly match those reconstructed from the patient CT scans, which were used in the simulations. This misalignment might have caused the differences in valve dynamics between the experiments and the simulations. For a visualisation of the misalignment we refer to Appendix B. We expect that the simulated and experimental results would agree better, when the calcification locations were more similar. Despite the differences in ejection period, the simulated mean transvalvular flows remained within the uncertainty bounds of the experimentally measured flows for d5, d10 and d20. Moreover, the simulations for the calcified valve successfully reproduced the overall valve behaviour under different dobutamine infusion rates. The maximum AVA values in the simulations agreed well with those in the experiments and fell within the measurement uncertainty of the experimental AVA. This observation indicates that the simulations with the calcified valve were able to accurately capture the valve opening behaviour. However, valve closure appeared to start earlier in the simulations, leading to the lower $Q_{\text{mean}}$ values.

To investigate whether the calibration of the shear modulus was dependent on the underlying data, we did a cross‐validation by calibrating the shear modulus on the d5 experiment. This calibration yielded a shear modulus of 75 kPa, only a 5% difference from the modulus (79 kPa) calibrated on d0. The discrepancies between mean transvalvular flows for the simulations and experiments were higher compared to those resulting from the calibration on d0, but still close to or within the uncertainty bounds. Moreover, the same positive relationship between mean transvalvular flow and pressure drop was observed in both simulations calibrated on d0 and d5. Therefore, we believe that the shear modulus calibration was sufficiently independent of the underlying data.

One notable discrepancy between the FSI simulations and the in vitro experiments, for both the non‐calcified and calcified valve, was observed. The simulations exhibited oscillations in flow during diastole, which were not present in the experimental flows. This discrepancy is probably caused by the fact that a rigid tube instead of a flexible tube representing the aorta was used, which will be detailed up on in the limitations section.

In short, some discrepancies were observed between the FSI model and the in vitro experiments regarding flow and valve dynamics. These differences could be attributed to inherent uncertainties in the FSI model as well as in the experiments. The deviations between simulation and experimental results were more pronounced in the calcified valve compared to the non‐calcified valve, which is expected as the inclusion of calcifications introduces additional complexity and uncertainty. Nevertheless, our results demonstrated that, after calibration of the material parameters, our FSI model can be used to simulate aortic valve fluid dynamics under different dobutamine infusion rates. This capability highlights the potential of our FSI model as a tool for non‐invasive dobutamine stress testing.

A study closely related to ours is that of Govindarajan et al. [18], in which a virtual stress test was simulated using a patient‐specific fluid–structure interaction (FSI) model with stepwise increasing left ventricular contraction. While their work provides valuable insights, our study extends this approach in several important ways. Most notably, we incorporated the closing behaviour of the aortic valve and simulated multiple cardiac cycles, which was not addressed in their simulations. Simulating multiple cardiac cycles enables the analysis of beat‐to‐beat variation and allows for the calculation of standard deviation and a range for metrics such as mean transvalvular flow. Note that the prescribed pressure signals in the simulations are periodic, but the resulting flows exhibit beat‐to‐beat variation induced by the stochastic nature of the simulations due to turbulence. An additional advantage is that pathophysiological conditions during diastole, such as aortic valve regurgitation, could be captured as well when modelling valve closure. Simulating valve closure and the diastolic phase of the cardiac cycle is challenging in FSI modelling. During closure, intersection of leaflets should be prevented with an appropriate contact algorithm. During diastole, the valve leaflets need to withstand the high aortic pressures pushing them towards the left ventricle. Despite these difficulties, we successfully simulated both valve closure and the complete diastolic phase over multiple cardiac cycles. Our model also accounts for patient‐specific calcification patterns, which have been shown to significantly influence aortic valve fluid dynamics [16, 17]. Lastly, unlike the study by Govindarajan et al., our FSI simulations were verified against experiments, adding additional trust to our findings.

Aortic valve FSI models have previously been verified against in vitro experiments by Sigüenza et al. [19]. However, their model was based on an idealised, healthy valve geometry. Our study extended the verification to a diseased, patient‐specific aortic valve. This important addition demonstrates the capability of FSI simulations to accurately capture the complex haemodynamics of pathological aortic valves, thereby supporting their potential use in clinical applications.

### 4.1 Limitations

The study by Sigüenza et al. [19] also highlights the first limitation of our work. Unlike their study, we did not have access to particle image velocimetry measurements, and therefore could not directly verify the simulated velocity fields against experimental flow data. However, clinical decision‐making typically relies on parameters such as pressure drop, aortic valve area (AVA), and in some cases, stroke volume. All of these quantities were measurable in our experimental setup, allowing comparison of the FSI simulations to both in vitro and clinical data. Therefore, we believe that our FSI model was verified to a degree that is sufficient for its intended clinical applications.

The second limitation of our study was that, in our simulations the aorta was simulated as a rigid tube, whereas in the experiments it was a flexible tube. The rigid tube likely contributed to the flow oscillations observed during diastole in the simulations, oscillations that were not present in the experimental data. The damping effect of arterial wall compliance has been previously demonstrated by Hsu et al. [34], who showed that elastic walls in FSI simulations of bio‐prosthetic valves effectively suppressed oscillations that were present in simulations with rigid walls. Moreover, the discrepancy between experimental and simulated diastolic flow behaviour has been reported in several other studies [35, 36]. Oliveira et al. argued that the set up in their study with pressure boundary conditions at the inflow and outflow boundaries, did not account for the complexity in the experiments, where connections and pumps dampen any oscillations in the flow rate. These findings suggest that incorporating a compliant aortic tube in the simulation, could help address the discrepancy. However, modelling deformable walls introduces extra computational time, complexity and uncertainty in the model. Since this complexity is not needed to compute the quantities of interest, that is, AVA and mean transvalvular flow, we opted to simulate a rigid tube for simplicity.

The third limitation is related to our choice of boundary conditions. Instead of prescribing the same flow waveform used to drive the gear pump in the experiments, we opted to prescribe the measured pressure drop across the valve at the inlet, with a zero‐pressure boundary condition at the outlet. Potential measurement uncertainty or errors in the pressure measurements could have caused the discrepancy in mean transvalvular flow observed between the experiment and the simulation for dobutamine infusion rate 10 μg/kg/min. Although we attempted to prescribe the flow directly, this led to numerical instability and mass conservation issues.

Finally, there are two important numerical limitations. The first one concerns mass conservation in the FSI simulations. Due to the methods employed by the LS‐DYNA solver, strict mass conservation was not enforced, resulting in a discrepancy between inflow and outflow rates. However, still the deviation in mean transvalvular flow (approximately 10%) computed with the inflow and outflow remained within the range of experimental uncertainty. Mass conservation could be improved by tightening the FSI coupling tolerance. However, this approach significantly increased computational costs, making the simulations impractically slow. Therefore, we accepted the discrepancy and proceeded with the more efficient setup. A more detailed analysis on mass conservation is provided in Appendix C.

The other numerical limitation is the time step size of Δt = 0.0002 s. When performing the same simulations with Δt = 0.0001 s, the resulting mean transvalvular flow and stroke volume are 10% lower. Although this decrease is substantial, it is considered acceptable since it falls within the uncertainty range of the experimental flow measurements.

### 4.2 Future Work

In this study, we verified our FSI model using only a single patient‐specific case. While this serves as an important proof of concept, further verification is necessary to establish the model's reliability for clinical decision‐making. Future research should therefore include additional mock‐loop experiments using silicone valve models derived from more patient anatomies. Moreover, it is important to emphasise that the in vitro experiments remain a simplification of reality, that is, the in vivo situation. The most critical discrepancy lies in the mismatch of Reynolds numbers: approximately 4000 for blood (transitional flow) versus 13,000 for water (turbulent flow). This difference significantly affects flow behaviour. For example, blood is expected to exhibit earlier flow separation and higher wall shear stresses compared to water. Consequently, an essential next step is to incorporate blood properties into the FSI model and perform validation against real clinical in vivo data.

After our FSI model is thoroughly verified and clinically validated, a logical next step would be to accelerate FSI simulations using machine learning techniques, thereby enhancing their feasibility for clinical application. For example, Pajaziti et al. [37] developed a method that enables rapid computation of 3D aortic pressure and velocity fields by combining machine learning with statistical shape modelling. Fresca et al. [38] proposed using deep learning‐based reduced‐order models for real‐time simulation of fluid flows, which they successfully applied to blood flow modelling in a cerebral aneurysm.

To integrate FSI simulations into clinical practice, future research should focus on developing efficient techniques to estimate leaflet stiffness and pre‐stress directly from data collected in clinical workflows. In our study, pre‐stress was not taken into account. Regarding the leaflet stiffness, we were able to calibrate the shear modulus of the valve by matching the simulated stroke volume with the experimental value. In a clinical setting, a similar approach could potentially be employed using stroke volume data obtained via Doppler echocardiography. However, this method requires multiple evaluations of the computationally expensive FSI model, resulting in substantial computational time. Therefore, a faster approach to estimate patient‐specific material properties and pre‐stress should be established. Existing approaches often involve solving inverse problems using clinical data, as comprehensively reviewed by Nolte et al. [39]. For instance, Bertoglio et al. [40] introduced a computationally efficient method for estimating arterial wall stiffness in FSI problems using reduced‐order unscented Kalman filtering. They later demonstrated its clinical applicability and verified the approach through an in vitro study [41]. Another promising technique is presented by Parikh et al., who employed inverse finite element analysis to estimate the mechanical properties of the aorta, while preserving pre‐stresses.

Moreover, the accuracy and reliability of patient‐specific FSI simulations heavily depend on the quality and availability of clinical data. Therefore, future research should explore how routinely acquired data from standard clinical protocols could be used in our FSI model. Currently our FSI model required CT imaging to reconstruct valve geometry and calcifications, (invasive) pressure measurements to define boundary conditions, and Doppler echocardiography to extract flow information and estimate material properties. However, CT images are typically only performed in patients already scheduled for valve replacement and are not part of the decision making protocol. Invasive pressure measurements are not part of standard clinical practice, and they are not desirable due to their invasive nature. Consequently, adapting the FSI model to rely solely on Doppler echocardiography data would be a valuable step towards integrating it into routine clinical workflows for aortic stenosis severity assessment.

To further demonstrate the clinical utility of our FSI model, particularly in assessing aortic stenosis (AS) severity, a promising future step would be to replicate a clinical trial in an in silico trial. For example, in the clinical study of Johnson et al. [4] pressure drop versus flow relationships were investigated in patients with severe AS. Additionally, Zelis et al. [5], and Eerdekens et al. [42], conducted clinical studies on evaluating the use of the stress aortic valve index (SAVI) in patients with moderate AS. Similar in silico trials could be performed using our FSI model. To this end, we could use the large dataset of 97 patient‐specific AS cases presented in our previous study and expand it further using our virtual cohort generator to create synthetic aortic valve geometries [43]. Synthetic calcification patterns could be generated using the machine learning approach of Oldenburg et al. [44]. This would enable a comprehensive investigation of the relationship between flow rate and pressure drop by simulating stepwise increases in flow conditions and we could compare it to the relations observed in the aforementioned clinical studies.

## 5 Conclusion

In this study, a patient‐specific FSI model of aortic stenosis was verified against an in vitro mock‐loop circulation experiment. The FSI model was capable of simulating six full cardiac cycles. The model incorporated patient‐specific calcification patterns and we successfully simulated four stepwise increasing flow conditions. Verification against experimental data from a mock‐loop circulation, representing the same patient‐specific anatomy, showed strong agreement in terms of mean transvalvular flow and aortic valve area. Before this model can be translated into clinical practice, two key challenges must be addressed: (1) the development of a robust method to estimate the material properties and pre‐stress of valve leaflets, and (2) verification and validation against a broader range of in vitro and clinical data, respectively. Despite these remaining steps, our work represents a valuable step towards the clinical implementation of FSI simulations. We have demonstrated the feasibility and potential use of our framework to serve as a robust, non‐invasive method to assess aortic stenosis severity and to support in clinical decision‐making on valve replacement.

## Funding

This work was supported by Horizon 2020 Framework Programme (101017578).

## Ethics Statement

The data used in this study were collected in Catharina Hospital, Eindhoven between February and October 2016. All subjects gave written informed consent as approved by the medical ethics committee of the hospital.

## Conflicts of Interest

The authors declare no conflicts of interest.

## Supporting information


**Movie S1:** Non‐calcified aortic valve in in vitro experiments (left) and FSI simulations (right) for dobutamine infusion rate 0 μg/kg/min.
**Movie S2:** Non‐calcified aortic valve in in vitro experiments (left) and FSI simulations (right) for dobutamine infusion rate 5 μg/kg/min.
**Movie S3:** Non‐calcified aortic valve in in vitro experiments (left) and FSI simulations (right) for dobutamine infusion rate 10 μg/kg/min.
**Movie S4:** Non‐calcified aortic valve in in vitro experiments (left) and FSI simulations (right) for dobutamine infusion rate 20 μg/kg/min.
**Movie S5:** Calcified aortic valve in in vitro experiments (left) and FSI simulations (right, calcifications in red) for dobutamine infusion rate 0 μg/kg/minR.
**Movie S6:** Calcified aortic valve in in vitro experiments (left) and FSI simulations (right, calcifications in red) for dobutamine infusion rate 5 μg/kg/min.
**Movie S7:** Calcified aortic valve in in vitro experiments (left) and FSI simulations (right, calcifications in red) for dobutamine infusion rate 10 μg/kg/min.
**Movie S8:** Calcified aortic valve in in vitro experiments (left) and FSI simulations (right, calcifications in red) for dobutamine infusion rate 20 μg/kg/min.

## Figures and Tables

**FIGURE A1:**
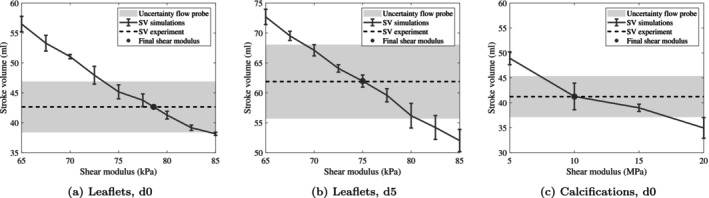
Stroke volume as a function of shear modulus of the leaflets for d0 (a) and d5 (b) and of the calcifications for d0 (c). The error bars represent the standard deviation over de cardiac cycles. The stroke volume and its uncertainty of the experiment are depicted in blue. The final chosen shear modulus is indicated with the red dot (79 kPa for the leaflets, and 10 MPa for the calcifications).

**FIGURE B1:**
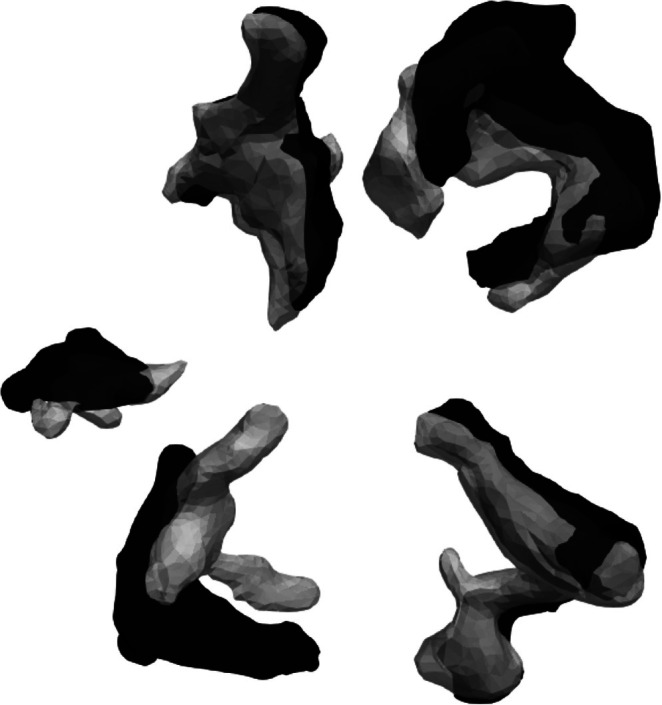
Alignment between calcifications in the silicone rubber valve models (black) and calcifications used in the simulations (white).

**FIGURE C1:**
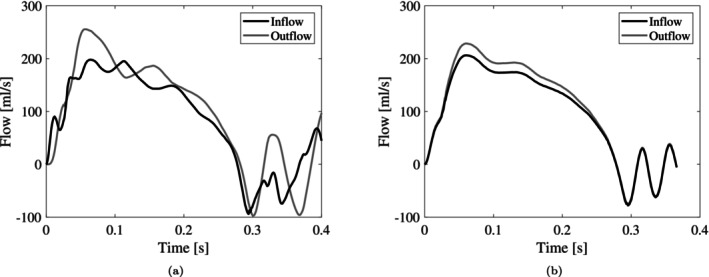
Simulated flow rates at the inflow and outflow boundaries for the default simulation (a) and with the FSI coupling tolerance reduced by half (b). Reduction of tolerance improved mass conservation.

## Data Availability

The data that support the findings of this study are openly available in 4TU.ResearchData at https://doi.org/10.4121/2915f548‐e8a2‐457e‐b1f8‐4151674f16c1.

## Appendix A

Shear Modulus Estimation.

FSI simulations were conducted for the baseline experiment d0, with the non‐calcified valve, across a range of shear moduli ranging from 65 to 85 kPa in 2.5 kPa increments, each over four complete cardiac cycles. For each simulation, stroke volume was calculated per cycle, and the mean and standard deviation were determined using the method described in Section 2.4. The results are shown in Figure A1a and compared to the experimentally measured stroke volume. To determine the most appropriate value for subsequent simulations, linear interpolation was performed between the two simulated stroke volumes that most closely matched the experimentally measured value. This interpolation yielded a shear modulus of 79 kPa, corresponding to a Young's modulus of 237 kPa. The interpolated stroke volume fell within the measurement accuracy (10%) of the ultrasonic flow probe. Additionally, we performed a cross‐validation by calibrating the leaflet shear modulus using experiment d5 (Figure A1b), which resulted in a value of 75 kPa, only a 5% difference compared to the modulus obtained from d0.

Using the shear modulus of the leaflets based on d0 (79 kPa), the shear modulus of the calcified deposits was determined by again running FSI simulations across a range of shear moduli from 5 to 20 MPa, in steps of 2.5 MPa. The shear modulus of 10 MPa, corresponding to a Young's modulus of 30 MPa, yielded the closest match with the experiments regarding stroke volume (Figure A1c) and was therefore used for the subsequent simulations.

## Appendix B

Alignment Calcifications In Vitro Experiments and FSI Simulations.

To visualise the alignment between the calcifications of the manually positioned silicone valve calcifications, and the calcifications reconstructed from the original CT scan, the silicone valve models were scanned using a micro‐CT scanner (Scanco Medical μCT100), and the calcifications were reconstructed. Figure B1 shows the overlay of the calcifications in the silicone valve model with those in the simulations. Notably, the upper left and lower right calcifications in the simulations appear closer to the centre of the valve, compared to those in the silicone valve model, which might have led to the altered valve dynamics.

## Appendix C

Mass Conservation.

An important limitation of the FSI simulations was the lack of strict mass conservation, since there was a discrepancy between the flows at the inflow and outflow boundaries (Figure C1a). This discrepancy likely arises from the solid solver occasionally relaxing the incompressibility constraint locally to facilitate numerical convergence. As a result, the inlet and outlet flows are not perfectly equal. To assess the impact of this limitation on our outputs of interest, we compared stroke volume and mean transvalvular flow derived from both inlet and outlet flow data. The stroke volume was 43 ± 1 mL (outflow boundary) versus 38 ± 1 mL (inflow boundary), yielding a 13% deviation. The mean transvalvular flow was 151 ± 2 mL/s (outflow boundary) versus 136 ± 2 mL/s (inflow boundary), corresponding to a 11% deviation. Although these deviations are non‐negligible, they are in the same order of magnitude as the experimental measurement uncertainty. Therefore, we consider the simulation results to be sufficiently accurate for the purpose of this study. Note that for the analyses in Section 3, the outlet flow was used. We also tried enhancing mass conservation by reducing the FSI coupling tolerance by half, which led to a noticeable improvement (Figure C1b). This adjustment resulted in a closer alignment between the inlet and outlet flow rates, which now follow each other more consistently and remain in phase. The deviation in stroke volume and transvalvular flow also showed slight improvements. Specifically, the stroke volume was 41 mL at the outflow boundary compared to 38 mL at the inflow boundary, corresponding to a deviation of 8%. Similarly, the mean transvalvular flow was 148 mL/s versus 135 mL/s at the inflow boundary, yielding a deviation of 10%. However, this reduction led to a substantial computational cost, increasing the simulation time from half a day to approximately 1 week per cardiac cycle. Therefore, we opted for the more computationally efficient simulation set up which still yielded results with acceptable accuracy with regards to mass conservation.

## References

[1] L. P. Fried , N. O. Borhani , P. Enright , et al., “The Cardiovascular Health Study: Design and Rationale,” Annals of Epidemiology 1, no. 3 (1991): 263–276. 1669507 10.1016/1047-2797(91)90005-w

[2] P. Collet , D. Foldager , G. Habib , and C. Hassager , “2021 Esc/Eacts Guidelines for the Management of Valvular Heart Disease,” European Journal of Cardio‐Thoracic Surgery 60 (2021): 727–800. 34453161 10.1093/ejcts/ezab389

[3] M.‐A. Clavel , J. Magne , and P. Pibarot , “Low‐Gradient Aortic Stenosis,” European Heart Journal 37, no. 34 (2016): 2645–2657. 27190103 10.1093/eurheartj/ehw096 PMC5030681

[4] N. P. Johnson , J. M. Zelis , P. A. Tonino , et al., “Pressure Gradient vs. Flow Relationships to Characterize the Physiology of a Severely Stenotic Aortic Valve Before and After Transcatheter Valve Implantation,” European Heart Journal 39, no. 28 (2018): 2646–2655. 29617762 10.1093/eurheartj/ehy126 PMC6055586

[5] J. M. Zelis , P. A. Tonino , D. T. Johnson , et al., “Stress Aortic Valve Index (Savi) With Dobutamine for Low‐Gradient Aortic Stenosis: A Pilot Study,” Structural Heart 4, no. 1 (2020): 53–61.

[6] L. G. Gilstrap , R. S. Bhatia , R. B. Weiner , and D. M. Dudzinski , “Dobutamine Stress Echocardiography: A Review and Update,” Research Reports in Clinical Cardiology 5 (2014): 69–81.

[7] M. R. Labrosse , C. J. Beller , M. Boodhwani , C. Hudson , and B. Sohmer , “Subject‐Specific Finite‐Element Modeling of Normal Aortic Valve Biomechanics From 3d+ t Tee Images,” Medical Image Analysis 20, no. 1 (2015): 162–172. 25476416 10.1016/j.media.2014.11.003

[8] M. Stevanella , E. Votta , M. Lemma , C. Antona , and A. Redaelli , “Finite Element Modelling of the Tricuspid Valve: A Preliminary Study,” Medical Engineering & Physics 32, no. 10 (2010): 1213–1223. 20869291 10.1016/j.medengphy.2010.08.013

[9] F. Borowski , M. Sämann , S. Pfensig , et al., “Fluid‐Structure Interaction of Heart Valve Dynamics in Comparison to Finite‐Element Analysis,” Current Directions in Biomedical Engineering 4, no. 1 (2018): 259–262.

[10] A. G. Kuchumov , A. Makashova , S. Vladimirov , V. Borodin , and A. Dokuchaeva , “Fluid–Structure Interaction Aortic Valve Surgery Simulation: A Review,” Fluids 8, no. 11 (2023): 295.

[11] J. De Hart , G. W. Peters , P. J. Schreurs , and F. P. Baaijens , “A Two‐Dimensional Fluid–Structure Interaction Model of the Aortic Value,” Journal of Biomechanics 33, no. 9 (2000): 1079–1088. 10854880 10.1016/s0021-9290(00)00068-3

[12] R. Van Loon , P. D. Anderson , J. De Hart , and F. P. Baaijens , “A Combined Fictitious Domain/Adaptive Meshing Method for Fluid–Structure Interaction in Heart Valves,” International Journal for Numerical Methods in Fluids 46, no. 5 (2004): 533–544.

[13] G. Luraghi , F. Migliavacca , and J. F. Rodriguez Matas , “Study on the Accuracy of Structural and Fsi Heart Valves Simulations,” Cardiovascular Engineering and Technology 9 (2018): 723–738. 30132282 10.1007/s13239-018-00373-3

[14] A. Morany , K. Lavon , R. Gomez Bardon , et al., “Fluid–Structure Interaction Modeling of Compliant Aortic Valves Using the Lattice Boltzmann Cfd and Fem Methods,” Biomechanics and Modeling in Mechanobiology 22, no. 3 (2023): 837–850. 36763197 10.1007/s10237-022-01684-0 PMC12077742

[15] G. B. K. Sundaram , K. R. Balakrishnan , and R. K. Kumar , “Aortic Valve Dynamics Using a Fluid Structure Interaction Model–the Physiology of Opening and Closing,” Journal of Biomechanics 48, no. 10 (2015): 1737–1744. 26058838 10.1016/j.jbiomech.2015.05.012

[16] L. Cai , Y. Hao , P. Ma , G. Zhu , X. Luo , and H. Gao , “Fluid‐Structure Interaction Simulation of Calcified Aortic Valve Stenosis,” Mathematical Biosciences and Engineering 19, no. 12 (2022): 13172–13192. 36654041 10.3934/mbe.2022616

[17] V. Govindarajan , A. Kolanjiyil , N. P. Johnson , H. Kim , K. B. Chandran , and D. D. McPherson , “Improving Transcatheter Aortic Valve Interventional Predictability via Fluid–Structure Interaction Modelling Using Patient‐Specific Anatomy,” Royal Society Open Science 9, no. 2 (2022): 211694. 35154799 10.1098/rsos.211694 PMC8826300

[18] V. Govindarajan , A. Kolanjiyil , C. Wanna , et al., “Biomechanical Evaluation of Aortic Valve Stenosis by Means of a Virtual Stress Test: A Fluid–Structure Interaction Study,” Annals of Biomedical Engineering 52, no. 2 (2024): 414–424. 37957528 10.1007/s10439-023-03389-6

[19] J. Sigüenza , D. Pott , S. Mendez , et al., “Fluid‐Structure Interaction of a Pulsatile Flow With an Aortic Valve Model: A Combined Experimental and Numerical Study,” International Journal for Numerical Methods in Biomedical Engineering 34, no. 4 (2018): e2945. 29181891 10.1002/cnm.2945

[20] DoD, “Modeling and Simulation (ms) Management,” 1994.

[21] S. Schlesinger , “Terminology for Model Credibility,” Simulation 32, no. 3 (1979): 103–104.

[22] W. L. Oberkampf and C. J. Roy , Verification and Validation in Scientific Computing (Cambridge University Press, 2010).

[23] J. M. Zelis , R. Meiburg , J. J. Roijen , et al., “3d‐Printed Stenotic Aortic Valve Model to Simulate Physiology Before, During, and After Transcatheter Aortic Valve Implantation,” International Journal of Cardiology 313 (2020): 32–34. 32380248 10.1016/j.ijcard.2020.04.087

[24] M. C. Geven , V. N. Bohté , W. H. Aarnoudse , et al., “A Physiologically Representative In Vitro Model of the Coronary Circulation,” Physiological Measurement 25, no. 4 (2004): 891–904. 15382829 10.1088/0967-3334/25/4/009

[25] Transonic Systems Inc , Tubing Flow (Clinical) Handbook: EC‐1‐hb, Rev A (Transonic Systems Inc, 2017).

[26] J. Donea , S. Giuliani , and J.‐P. Halleux , “An Arbitrary Lagrangian‐Eulerian Finite Element Method for Transient Dynamic Fluid‐Structure Interactions,” Computer Methods in Applied Mechanics and Engineering 33, no. 1–3 (1982): 689–723.

[27] R. Codina , “Pressure Stability in Fractional Step Finite Element Methods for Incompressible Flows,” Journal of Computational Physics 170, no. 1 (2001): 112–140.

[28] S. R. Idelsohn , F. Del Pin , R. Rossi , and E. Oñate , “Fluid–Structure Interaction Problems With Strong Added‐Mass Effect,” International Journal for Numerical Methods in Engineering 80, no. 10 (2009): 1261–1294.

[29] J. W. Deardorff , “A Numerical Study of Three‐Dimensional Turbulent Channel Flow at Large Reynolds Numbers,” Journal of Fluid Mechanics 41, no. 2 (1970): 453–480.

[30] C. Chnafa , S. Mendez , and F. Nicoud , “Image‐Based Large‐Eddy Simulation in a Realistic Left Heart,” Computers & Fluids 94 (2014): 173–187.

[31] S. B. Pope , “Ten Questions Concerning the Large‐Eddy Simulation of Turbulent Flows,” New Journal of Physics 6, no. 1 (2004): 35.

[32] J. Bonet and A. Burton , “A Simple Average Nodal Pressure Tetrahedral Element for Incompressible and Nearly Incompressible Dynamic Explicit Applications,” Communications in Numerical Methods in Engineering 14, no. 5 (1998): 437–449.

[33] M. A. Puso and T. A. Laursen , “A Mortar Segment‐To‐Segment Contact Method for Large Deformation Solid Mechanics,” Computer Methods in Applied Mechanics and Engineering 193, no. 6–8 (2004): 601–629.

[34] M.‐C. Hsu , D. Kamensky , Y. Bazilevs , M. S. Sacks , and T. J. Hughes , “Fluid–Structure Interaction Analysis of Bioprosthetic Heart Valves: Significance of Arterial Wall Deformation,” Computational Mechanics 54, no. 4 (2014): 1055–1071. 25580046 10.1007/s00466-014-1059-4 PMC4286305

[35] H. L. Oliveira , G. C. Buscaglia , R. R. Paz , et al., “Three‐Dimensional Fluid–Structure Interaction Simulation of the Wheatley Aortic Valve,” International Journal for Numerical Methods in Biomedical Engineering 40, no. 2 (2024): e3792. 38010884 10.1002/cnm.3792

[36] K. Baylous , B. Kovarovic , R. R. Paz , et al., “Thrombogenic Risk Assessment of Transcatheter Prosthetic Heart Valves Using a Fluid‐Structure Interaction Approach,” Computer Methods and Programs in Biomedicine 257 (2024): 108469. 39461118 10.1016/j.cmpb.2024.108469 PMC12085998

[37] E. Pajaziti , J. Montalt‐Tordera , C. Capelli , et al., “Shape‐Driven Deep Neural Networks for Fast Acquisition of Aortic 3D Pressure and Velocity Flow Fields,” PLoS Computational Biology 19, no. 4 (2023): e1011055. 37093855 10.1371/journal.pcbi.1011055 PMC10159343

[38] S. Fresca and A. Manzoni , “Real‐Time Simulation of Parameter‐Dependent Fluid Flows Through Deep Learning‐Based Reduced Order Models,” Fluids 6, no. 7 (2021): 259.

[39] D. Nolte and C. Bertoglio , “Inverse Problems in Blood Flow Modeling: A Review,” International Journal for Numerical Methods in Biomedical Engineering 38, no. 8 (2022): e3613. 35526113 10.1002/cnm.3613 PMC9541505

[40] C. Bertoglio , P. Moireau , and J.‐F. Gerbeau , “Sequential Parameter Estimation for Fluid–Structure Problems: Application to Hemodynamics,” International Journal for Numerical Methods in Biomedical Engineering 28, no. 4 (2012): 434–455. 25365657 10.1002/cnm.1476

[41] C. Bertoglio , D. Barber , N. Gaddum , et al., “Identification of Artery Wall Stiffness: In Vitro Validation and In Vivo Results of a Data Assimilation Procedure Applied to a 3D Fluid–Structure Interaction Model,” Journal of Biomechanics 47, no. 5 (2014): 1027–1034. 24529756 10.1016/j.jbiomech.2013.12.029

[42] R. Eerdekens , P. Tonino , J. Zelis , et al., “Rationale and Design of Savi‐Aos: A Physiologic Study of Patients With Symptomatic Moderate Aortic Valve Stenosis and Preserved Left Ventricular Ejection Fraction,” IJC Heart & Vasculature 41 (2022): 101063. 35663622 10.1016/j.ijcha.2022.101063 PMC9157233

[43] S. Verstraeten , M. Hoeijmakers , P. Tonino , et al., “Generation of Synthetic Aortic Valve Stenosis Geometries for In Silico Trials,” International Journal for Numerical Methods in Biomedical Engineering 40, no. 1 (2024): e3778. 37961993 10.1002/cnm.3778

[44] J. Oldenburg , F. Borowski , L. Supp , A. Öner , K.‐P. Schmitz , and M. Stiehm , “Generation of Shape Models of Calcified Tavr Populations for Solid Mechanics Simulations by Means of Deep Learning,” in Current Directions in Biomedical Engineering, vol. 10 (De Gruyter, 2024), 469–472.

