# Supplementary material for: Verification of a Fluid–Structure Interaction Model for Aortic Stenosis Through Comparison With In Vitro Experiments
Source: Int J Numer Method Biomed Eng. 2026 Mar 31;42(4):e70169. doi: 10.1002/cnm.70169 (PMC13039782; doi:10.1002/cnm.70169)
Supplement: Supplementary file 1 — Movie S1: Non‐calcified aortic valve in in vitro experiments (left) and FSI simulations (right) for dobutamine infusion rate 0 μg/kg/min. Movie S2: Non‐calcified aortic valve in in vitro experiments (left) and FSI simulations (right) for dobutamine infusion rate 5 μg/kg/min. Movie S3: Non‐calcified aortic valve in in vitro experiments (left) and FSI simulations (right) for dobutamine infusion rate 10 μg/kg/min. Movie S4: Non‐calcified aortic valve in in vitro experiments (left) and FSI simulations (right) for dobutamine infusion rate 20 μg/kg/min. Movie S5: Calcified aortic valve in in vitro experiments (left) and FSI simulations (right, calcifications in red) for dobutamine infusion rate 0 μg/kg/minR. Movie S6: Calcified aortic valve in in vitro experiments (left) and FSI simulations (right, calcifications in red) for dobutamine infusion rate 5 μg/kg/min. Movie S7: Calcified aortic valve in in vitro experiments (left) and FSI simulations (right, calcifications in red) for dobutamine infusion rate 10 μg/kg/min. Movie S8: Calcified aortic valve in in vitro experiments (left) and FSI simulations (right, calcifications in red) for dobutamine infusion rate 20 μg/kg/min. [file CNM-42-e70169-s001.docx]

# Supplementary movies

**Movie 1:** non-calcified aortic valve in in vitro experiments (left) and FSI simulations (right) for dobutamine infusion rate 0 $\mu$g/kg/min

**Movie 2:** non-calcified aortic valve in *in vitro* experiments (left) and FSI simulations (right) for dobutamine infusion rate 5 $\mu$g/kg/min

**Movie 3:** non-calcified aortic valve in *in vitro* experiments (left) and FSI simulations (right) for dobutamine infusion rate 10 $\mu$g/kg/min

**Movie 4:** non-calcified aortic valve in *in vitro* experiments (left) and FSI simulations (right) for dobutamine infusion rate 20 $\mu$g/kg/min

**Movie 5:** calcified aortic valve in *in vitro* experiments (left) and FSI simulations (right, calcifications in red) for dobutamine infusion rate 0 $\mu$g/kg/min

**Movie 6:** calcified aortic valve in *in vitro* experiments (left) and FSI simulations (right, calcifications in red) for dobutamine infusion rate 5 $\mu$g/kg/min

**Movie 7:** calcified aortic valve in *in vitro* experiments (left) and FSI simulations (right, calcifications in red) for dobutamine infusion rate 10 $\mu$g/kg/min

**Movie 8:** calcified aortic valve in *in vitro* experiments (left) and FSI simulations (right, calcifications in red) for dobutamine infusion rate 20 $\mu$g/kg/min
